# Different Catalytic Mechanisms in Mammalian Selenocysteine- and Cysteine-Containing Methionine-*R*-Sulfoxide Reductases

**DOI:** 10.1371/journal.pbio.0030375

**Published:** 2005-11-08

**Authors:** Hwa-Young Kim, Vadim N Gladyshev

**Affiliations:** **1**Department of Biochemistry, University of Nebraska, Lincoln, Nebraska, United States of America; University of MichiganUnited States of America

## Abstract

Selenocysteine (Sec) is found in active sites of several oxidoreductases in which this residue is essential for catalytic activity. However, many selenoproteins have fully functional orthologs, wherein cysteine (Cys) occupies the position of Sec. The reason why some enzymes evolve into selenoproteins if the Cys versions may be sufficient is not understood. Among three mammalian methionine-*R*-sulfoxide reductases (MsrBs), MsrB1 is a Sec-containing protein, whereas MsrB2 and MsrB3 contain Cys in the active site, making these enzymes an excellent system for addressing the question of why Sec is used in biological systems. In this study, we found that residues, which are uniquely conserved in Cys-containing MsrBs and which are critical for enzyme activity in MsrB2 and MsrB3, were not required for MsrB1, but increased the activity of its Cys mutant. Conversely, selenoprotein MsrB1 had a unique resolving Cys reversibly engaged in the selenenylsulfide bond. However, this Cys was not necessary for activities of either MsrB2, MsrB3, or the Cys mutant of MsrB1. We prepared Sec-containing forms of MsrB2 and MsrB3 and found that they were more than 100-fold more active than the natural Cys forms. However, these selenoproteins could not be reduced by the physiological electron donor, thioredoxin. Yet, insertion of the resolving Cys, which was conserved in MsrB1, into the selenoprotein form of MsrB3 restored the thioredoxin-dependent activity of this enzyme. These data revealed differences in catalytic mechanisms between selenoprotein MsrB1 and non-selenoproteins MsrB2 and MsrB3, and identified catalytic advantages and disadvantages of Sec- and Cys-containing proteins. The data also suggested that Sec- and Cys-containing oxidoreductases require distinct sets of active-site features that maximize their catalytic efficiencies and provide strategies for protein design with improved catalytic properties.

## Abstract 2

Altering cysteine-containing residues in a family of oxidoreductases reveals the role of selenocysteine in influencing the catalytic mechanism.

## Introduction

Several oxidoreductases, such as glutathione peroxidase, thioredoxin (Trx) reductase, and methionine sulfoxide (Met-SO) reductase, have selenocysteine (Sec) in their active sites [1–3]. These selenoproteins are typically 100- to 1,000-fold more active than their cysteine (Cys) mutants. This high catalytic activity of Sec-containing enzymes has been regarded as a key reason why Sec is used in biological systems [4–7]. However, some selenoproteins have orthologs, in which Cys is used in place of Sec, and which are as catalytically competent as selenoproteins [8].

Met-SO reductases catalyze the reduction of free and protein-bound Met-SO back to methionine in the presence of Trx (reviewed in [9]). There are two distinct classes of Met-SO reductases; MsrA is specific for the *S* isomer of Met-SO, whereas methionine-*R*-sulfoxide reductase (MsrB) can reduce only the *R* form of this compound. Met-SO reduction is thought to be an important protein-repair pathway that provides protection against oxidative stress, regulates protein function, and delays aging (reviewed in [10–13]). Most organisms from bacteria to humans employ Met-SO reduction systems to repair oxidized methionine residues. Human and mouse genomes possess a single MsrA and three MsrB genes that code for MsrB1, MsrB2, and MsrB3 (reviewed in [14]). Among the three mammalian MsrBs, MsrB1 (also known as selenoprotein R or X) has Sec in its active site [3,15], whereas MsrB2 (also known as CBS-1) and MsrB3 contain Cys in place of Sec [16–18]. The three MsrBs are present in different cellular compartments. MsrB1 is in the cytosol and nucleus, MsrB2 is in the mitochondria, and MsrB3 is either in the endoplasmic reticulum or in the mitochondria depending on alternatively spliced forms and on the organism in which it occurs [18,19].

A recent study by Gromer et al. addressed the question of why Sec is used in Trx reductases [8]. These authors showed that serine (Ser) residues that flank the catalytic Cys–Cys motif at the C-terminus of the protein provide necessary adjustments that make a non-selenoprotein *Drosophila* Trx reductase as active as its mammalian selenoprotein counterparts. This study suggested that Sec is not necessarily required for efficient catalysis by Trx reductases. However, the selenoenzymes provide advantages in terms of a broader range of substrates and increased flexibility in microenvironmental conditions in the active sites.

We previously expressed, in *Escherichia coli,* a recombinant selenoprotein form of MsrB1, which has four mutant residues (S99R, S100L, K102G, and F103P) [18]. These four mutations introduced a selenocysteine insertion sequence (SECIS) element downstream of the Sec-encoding UGA codon. This recombinant selenoprotein exhibited ∼1,000-fold higher enzyme activity than its Cys counterpart, indicating the essential role of Sec in this enzyme. However, the activities of recombinant MsrB2 and MsrB3 were ∼200-fold higher than that of the Cys-containing MsrB1 mutant, and only ∼4-fold lower than that of the recombinant selenoprotein MsrB1.

The fact that mammals have one selenoprotein (MsrB1) and two Cys-containing homologs (MsrB2 and MsrB3), all with similar catalytic efficiencies, allows these enzymes to be used to address the question of why Sec is used in biological systems. In the present study, we performed extensive mutational analyses to better understand the catalytic advantages and disadvantages that Sec and Cys provide for MsrB function. We demonstrated differences in catalytic mechanisms between selenoprotein MsrB1 and non-selenoproteins MsrB2 and MsrB3, and found that Sec- and Cys-containing enzymes require different sets of features in the active site to maximize their activities.

## Results/Discussion

### Three Residues Conserved in Cys-Containing MsrBs but Absent in Selenoprotein MsrB1

**Figure 1:**
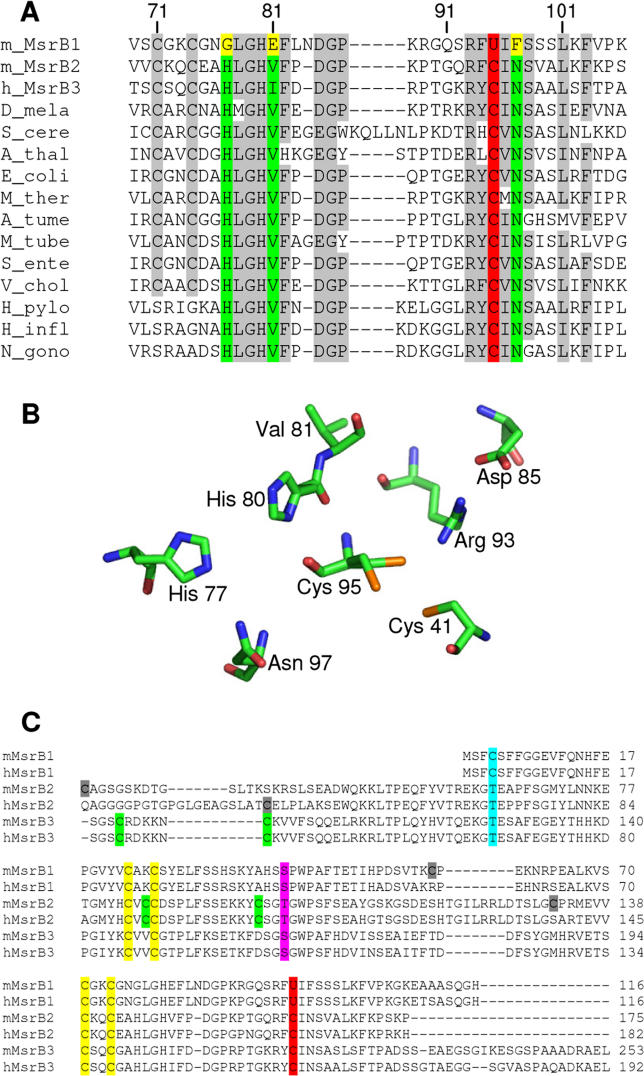
Conserved Features in MsrB Sequences and Structures (A) Alignment of MsrB sequences corresponding to the active sites in these proteins. Catalytic Cys (C) and Sec (U) residues are shown in red. The conserved His, Val/Ile, and Asn residues in Cys-containing proteins are indicated in green, whereas the corresponding residues (Gly, Glu, and Phe, respectively) in selenoprotein MsrB1 are highlighted in yellow. Gray shows other conserved residues. Numbering of amino acids is shown above the sequences and is based on the mouse MsrB1 sequence. (B) Structure of the active site of *N. gonorrhoeae* MsrB domain (1L1D). For convenience, some active-site residues are not shown. Numbering of amino acids is based on the mouse MsrB1 sequence. The Cys41 is the resolving Cys. It is absent in all mammalian MsrBs. (C) Alignment of mouse and human MsrB sequences. Catalytic Cys (C) and Sec (U) residues are shown in red. Pink indicates Ser and Thr residues that replace the conserved resolving Cys residues in many MsrB proteins. Zinc-coordinating Cys residues are shown in yellow. Other conserved Cys residues in each mouse and human MsrB sequence are indicated in green, whereas non-conserved Cys residues are shown in gray. Residues corresponding to Cys4 in MsrB1 are highlighted in turquoise.

Multiple-sequence alignment of available MsrB sequences revealed three highly conserved residues, which are present in most MsrBs, but are absent in selenoprotein MsrB1 sequences (Figure 1A). The first residue corresponded to Gly77 in mouse MsrB1 (hereinafter, numbering of amino acids is based on the MsrB1 sequence, unless noted otherwise). This residue was replaced with His in all Cys-containing MsrBs including mammalian MsrB2 and MsrB3. The second residue was Glu81 in selenoprotein MsrB1. All other MsrBs had Val or Ile in its place. The third residue was Phe97 in MsrB1. This residue was replaced with Asn in Cys-containing MsrBs. MsrBs have a wide distribution, but Sec-containing MsrBs are present only in vertebrates and co-occur in these organisms with Cys-containing MsrBs, consistent with recent evolution of the selenoprotein forms from animal Cys-containing MsrBs. This sequence analysis (Figure 1) raised questions as to why the selenoprotein MsrB1 has different residues at the three positions highly conserved in Cys-containing enzymes and what the roles of these residues are in the catalytic function of selenoprotein and non-selenoprotein MsrBs.

### Different Sets of Active-Site Features in Sec-Containing MsrB1 and Cys-Containing MsrB2 and MsrB3

**Figure 2:**
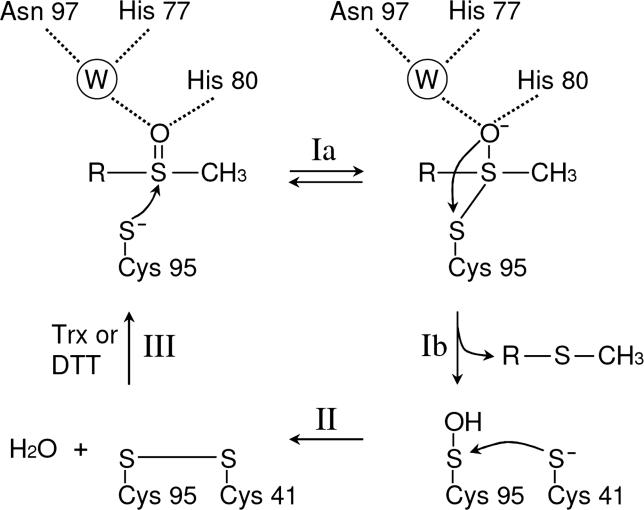
Schematic Representation of the Reaction Mechanism of *N. gonorrhoeae* MsrB Numbering of amino acids is based on the mouse MsrB1 sequence. A water molecule is indicated as W. This reaction mechanism was adapted from Lowther et al. [20]. The nucleophilic attack by Cys95 on sulfoxide moiety of the substrate results in a trigonal-bipyramidal intermediate (Ia), followed by the formation of the sulfenic acid intermediate of Cys95 and the release of methionine (Ib). The resolving Cys41 attacks the sulfenic acid intermediate of Cys95 to form a disulfide bond (II). The disulfide bond is then reduced by Trx in vivo or by DTT in vitro and the active site is returned to the fully reduced state (III).

A crystal structure of an MsrB domain of *Neisseria gonorrhoeae* PilB has been reported [20]. This domain corresponds to a Cys-containing MsrB. pKa values of Cys thiols are typically around 8.3, unless adjusted by microenvironmental conditions, whereas the selenol group of Sec is fully ionized at physiological pH owing to its low pKa value of 5.2 [4,5]. The *N. gonorrhoeae* MsrB structure suggested that the catalytic Cys95 nucleophile is activated by a Cys95–Arg93–Asp85 triad [20] (Figure 1B), which is conserved in all MsrBs except for replacement of Asp with Glu in some homologs. It was also proposed, based on the *N. gonorrhoeae* MsrB structure, that His77 and Asn97 form hydrogen bonds with a water molecule, which in turn interacts with the oxygen atom of the sulfoxide moiety of the substrate [20] (Figure 2). This hydrogen-bond network may stabilize the intermediate in the reaction [20]. Thus, the residues conserved in the Cys-containing MsrBs, but absent in selenoprotein MsrBs (see Figure 1), are part of the active site.

To further examine the location and function of active-site residues (Figure 1C), we developed molecular models of mouse MsrB1, mouse MsrB2, and human MsrB3 (Figure S1). The locations of the three residues conserved in Cys-containing MsrBs were similar to those in *N. gonorrhoeae* MsrB (Figure 1B). Therefore, we initially hypothesized that His77, Val/Ile81, and Asn97 in Cys-containing enzymes assist the catalytic Cys by stabilizing the thiolate, whereas Sec in the selenoenzyme MsrB1 would not need the assistance of these residues owing to its high reactivity and low pKa.

### Impairment of Selenoprotein MsrB1 Activity by the Residues Conserved in Cys-Containing MsrBs

To test the roles of these three residues in catalysis by selenoprotein MsrB1, we expressed a C-terminal His-tagged Sec-containing MsrB1 in mammalian cells from a construct containing a natural SECIS element in the 3′-untranslated region, and purified the protein by affinity chromatography. We also made a series of mutant constructs in which Gly77, Glu81, and Phe97 were mutated, individually or in combination, to His, Val, and Asn, respectively. The proteins were then assayed for dithiothreitol (DTT)- and Trx-dependent Met-SO reduction. If Sec did not require assistance from the residues which are specific for Cys-containing MsrBs, the single, double, or triple mutations involving G77H, E81V, and F97N would not be able to stimulate MsrB1 activity. Indeed, we found that single G77H, E81V, and F97N mutants exhibited 9-, 121-, and 26-fold lower activity, respectively, than the wild-type form in the DTT-dependent reaction (Table 1). The double G77H/G97N mutant had 15-fold lower activity than the wild-type enzyme, and the triple G77H/E81V/F97N mutant was completely inactive. The effects of these mutations on the Trx-dependent activity were even more severe (Table 1). Thus, the three residues conserved in Cys-containing MsrBs were not required for activity of the selenoprotein MsrB1 form and, in fact, were detrimental to its enzymatic function.

**Table 1:**
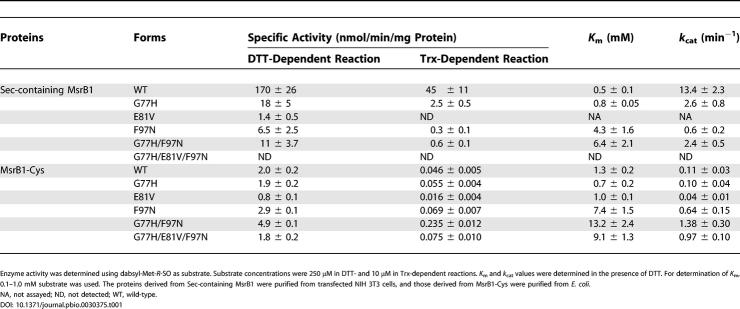
Specific Activity and Kinetic Constants of Wild-Type and Mutant Forms of Mouse MsrB1

### Increased Activity of the Cys Mutant of MsrB1 by Introducing Residues Conserved in Cys-Containing MsrBs

We examined the effects of mutations of Gly77, Glu81, and Phe97 on the catalytic activity of the Cys mutant of MsrB1 (MsrB1-Cys; i.e., Cys95 in place of Sec95). As shown in Table 1, the specific activity of the G77H version of MsrB1-Cys was similar to that of the original MsrB1-Cys in both DTT- and Trx-dependent reactions. However, the replacement of Phe97 with Asn (F97N mutant) increased the enzyme activity by 1.5-fold in both assays. Moreover, the double G77H/F97N mutant exhibited 2.5- and 5-fold higher activity in DTT- and Trx-dependent reactions, respectively, than MsrB1-Cys, indicating that these two residues synergistically increased enzyme activity. The *k*_cat_ values of F97N and G77H/F97N mutants were 6- and 13-fold higher, respectively, compared to that of MsrB1-Cys. Similar to the proposed role for His77 and Asn97 in the hydrogen-bond network in the active site of *N. gonorrhoeae* MsrB, these mutations (G77H and F97N) may have generated a hydrogen-bond network in the active site of MsrB1-Cys to assist the catalytic function of Cys95. Thus, the effects of G77H and F97N mutations in MsrB1-Cys are clearly in contrast to those observed in Sec-containing MsrB1. The third mutation, E81V, in contrast to expectations, decreased the MsrB1-Cys activity by 2.5-fold, and it also decreased the activity of the triple mutant (G77H/E81V/F97N) by 3-fold compared to the activity of the double mutant (G77H/F97N).

### Requirement of Residues Uniquely Conserved in Cys-Containing MsrBs for Catalytic Activity of These Enzymes

To investigate the roles of the three conserved residues in catalysis by Cys-containing MsrBs, we mutated these amino acids in mouse MsrB2 and human MsrB3 to those found in MsrB1. Table 2 summarizes specific activities of wild-type and various mutants of these enzymes. The V81E mutant of MsrB2 showed a 2-fold lower activity than the wild-type protein, and the activity of the H77G mutant was decreased 50-fold. The N97F mutation had the most dramatic effect, as activity of this protein was decreased 7.7 × 10^3^-fold in the presence of DTT, and was not detectable in the Trx-dependent reaction. Corresponding mutations in MsrB3 had similar effects (Table 2). Taken together, the data suggest that any changes in residues uniquely conserved in Cys-containing MsrBs to those present in selenoprotein MsrB resulted in low catalytic activity. Among these residues, Asn had the most dramatic effect.

**Table 2:**
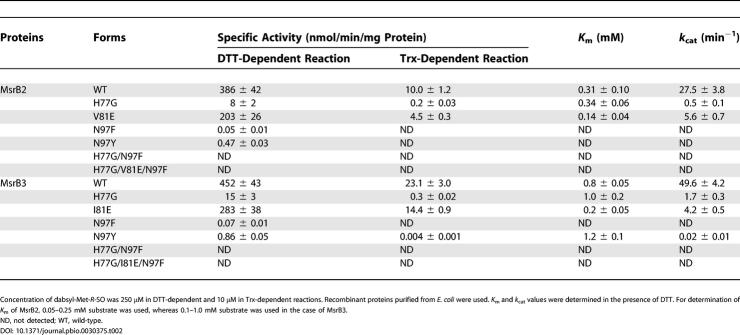
Specific Activities and Kinetic Constants of Wild-Type and Mutant Forms of Mouse MsrB2 and Human MsrB3

As discussed above, Asn likely participates in the hydrogen-bond network and is involved in binding of the substrate or intermediate in the reaction (see Figure 2). It is possible that substitution of Asn with Phe (as in MsrB1) disrupts this network. To test this idea further, we generated mutants of both MsrB2 and MsrB3 in which Asn was replaced by Tyr. This mutation increased the enzyme activity 10-fold compared to the Phe mutants. However, the Tyr mutants of MsrB2 and MsrB3 still exhibited 500- to 800-fold lower activity than the wild-type proteins. In our structural models of MsrB2 and MsrB3, Asn is located on the bottom of the catalytic pocket (Figure S1A). Thus, it is possible that substitution of Asn with a bulky aromatic amino acid, Phe, might not only disrupt the hydrogen-bond network in the active site but might also interfere with the accessibility of the substrate to the active site.

We determined *K*_m_ values of wild-type and mutant forms of all three MsrBs in the DTT-dependent reaction (see Tables 1 and 2). *K*_m_ values for dabsyl-Met-*R*-SO were 0.5 mM and 0.8 mM for the wild-type MsrB1 and the corresponding G77H mutant, respectively. *K*_m_ values for F97N and G77H/F97N mutants (4.3 and 6.4 mM, respectively) were significantly increased. *K*_m_ values for G77H and E81V forms of MsrB1-Cys (0.7 and 1.0 mM, respectively) were similar to that for the wild-type MsrB1-Cys (1.3 mM). In contrast, *K*_m_ values for F97N, G77H/F97N, and G77H/E81V/F97N mutants were much higher (6- to 10-fold) than that for MsrB1-Cys. These data, along with the observation of the increased activity of the F97N mutant, suggest that the F97N mutation increased the enzyme activity of MsrB1-Cys at higher concentrations of the substrate. *K*_m_ values for wild-type MsrB2 and MsrB3 were 0.31 and 0.8 mM, respectively. Several mutations, including H77G in MsrB2 and H77G or N97Y in MsrB3, did not change *K*_m_ values significantly. However, the V81E mutation in MsrB2, and the I81E mutation in MsrB3, lowered *K*_m_ values 2- and 4-fold, respectively.

### Increased Activity of Selenoprotein Forms of MsrB2 and MsrB3 Compared with the Natural Cys-Containing Enzymes in the DTT-Dependent Reaction

To further address the role of Sec in MsrBs, we developed a selenoprotein form of MsrB3. To prepare this protein, we inserted a SECIS element of mouse MsrB1 downstream of the stop codon, replaced the Cys95 codon with TGA, and introduced a His tag at the C-terminus for affinity isolation (seleno-MsrB3/C95U construct). We also generated single N97F and triple H77G/I81E/N97F mutant forms of seleno-MsrB3/C95U. The constructs were transfected into NIH 3T3 cells, and the seleno-MsrB3 proteins were purified from these cells by affinity chromatography. Remarkably, seleno-MsrB3/C95U had a 126-fold higher activity compared to the wild-type protein in the DTT-dependent reaction (Table 3). The fact that a simple mutation of the catalytic Cys to Sec could improve the enzyme activity so dramatically over that of the natural enzyme argues for an inherent catalytic advantage that may be provided by Sec over Cys in certain oxidoreductases.

**Table 3:**
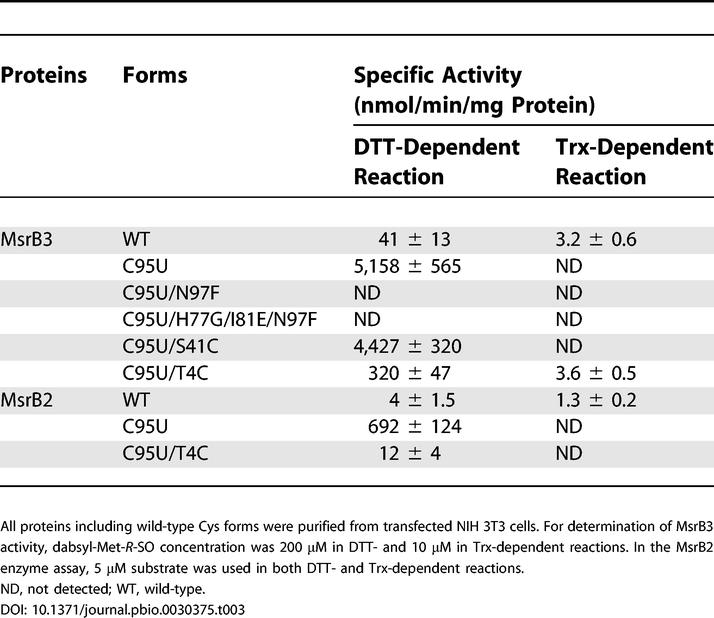
Specific Activities of Wild-Type and Selenoprotein Forms of MsrB3 and MsrB2

Further experiments revealed that neither single N97F nor triple H77G/I81E/N97F mutants of seleno-MsrB3/C95U were active, indicating that Asn97 was critical for the MsrB3 activity even in the selenoprotein form. As discussed above, it is possible that the presence of Phe97 in these mutants blocked the access of the substrate to the active site.

We similarly prepared the selenoprotein form of MsrB2. This protein, seleno-MsrB2/C95U, showed a 173-fold higher activity than the natural Cys-containing form in the DTT-dependent reaction (Table 3). The fact that substitution of Cys with Sec in MsrB2 and MsrB3 increased MsrB catalytic activity so markedly is in contrast to the observation that the activity of the Sec mutant of *Drosophila* Trx reductase did not change significantly compared to that of the natural Cys-containing protein [8].

Taken together, our data revealed that (i) His77 and Asn97 are required in Cys-containing MsrBs, and that these residues could even increase the activity of the MsrB1-Cys mutant; (ii) Gly77, Glu81, and Phe97 are required in selenoprotein MsrB1; and (iii) Sec per se could increase the activity of Cys-containing MsrBs.

### Difference in Trx Dependency between Selenoprotein and Non-Selenoprotein MsrBs

The reaction mechanism of MsrA is well understood [21–25]. It has been proposed that the catalytic mechanism of MsrBs from *Drosophila melanogaster, N. gonorrhoeae,* and *N. meningitides* [20,26–28] is similar to that of MsrA, even though MsrA and MsrB are structurally unrelated enzymes [20,22–24,29]. The previously proposed reaction mechanism of MsrB includes three steps (Figure 2): (i) an attack by the catalytic Cys on the sulfoxide moiety of the substrate with the formation of a sulfenic acid intermediate and the concomitant release of methionine; (ii) formation of an intramolecular disulfide bond between catalytic and resolving Cys residues; and (iii) reduction of the disulfide by Trx, a natural electron donor, or by DTT (which can serve as reductant in in vitro assays). DTT could also directly reduce the sulfenic acid intermediate.

Multiple-sequence alignment revealed that the resolving Cys41 (see Figure 1B) is conserved in only ∼60% of known MsrBs. The remaining ∼40% of MsrBs, including all three mammalian MsrBs, do not have this resolving Cys (Figure 1C). In addition, *Mycoplasma pulmonis* and *Vibrio cholerae* MsrBs have only a single Cys in their sequences, and several enzymes including that from *Mesorhizobium loti* have only a single Cys besides zinc-coordinating Cys residues. Two possible alternative reaction mechanisms can be postulated for MsrBs that lack the conserved resolving Cys, including (i) a direct reduction of the sulfenic acid intermediate by Trx (or other electron donors); and (ii) the use of an alternative resolving Cys to generate the intramolecular disulfide. The latter mechanism has been reported in a recent study [30], which identified an alternative resolving Cys in *Xanthomonas campestris* MsrB. However, this Cys is absent in mammalian MsrBs. We also analyzed mammalian MsrB1, MsrB2, and MsrB3 by gel filtration and found that these enzymes were monomeric (data not shown). Therefore, the reversible intermolecular disulfide or selenenylsulfide bond is not likely in these proteins.

As shown in Figure 1C, mouse MsrB1 contains six Cys residues (Cys4, Cys23, Cys26, Cys58, Cys71, and Cys74). Four of these residues (Cys23, Cys26, Cys71, and Cys74) are organized in two CxxC motifs and are involved in zinc coordination [26]. Cys58 is not conserved in several other mammalian MsrB1s, including human MsrB1.

**Figure 3:**
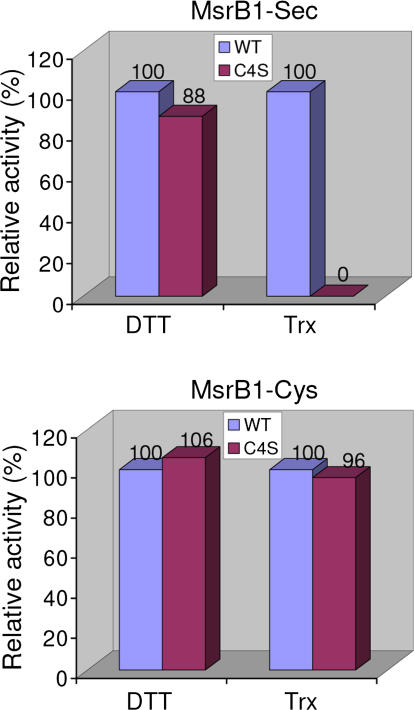
Cys4 Is Required for Trx-Dependent Reduction of Sec-Containing MsrB1 Catalytic activities of C4S mutant forms of selenoprotein MsrB1 (shown as MsrB1-Sec in the figure) and MsrB1-Cys are shown as compared to the activities of the corresponding proteins containing natural Cys4. Dabsyl-Met-*R*-SO substrate concentration was 250 μM in DTT- and 10 μM in Trx-dependent reactions. Sec-containing MsrB1 and its C4S mutant forms were purified from transfected NIH 3T3 cells, whereas MsrB1-Cys and its C4S mutant were obtained from *E. coli*. No activity of the C4S mutant of Sec-containing MsrB1 was detected in the Trx-dependent reaction.

Based on these observations, we hypothesized that the only remaining Cys in MsrB1, Cys4 (Figure 1C), is a candidate resolving residue that forms an intramolecular selenenylsulfide bond with the catalytic Sec95. To test this idea, we mutated this residue to Ser in both Sec-containing MsrB1 and MsrB1-Cys, and determined catalytic activities of the C4S mutants and corresponding wild-type enzymes in DTT- and Trx-dependent reactions. This mutation did not change the DTT-dependent activity of the selenoprotein (Figure 3). However, in the presence of Trx, the C4S mutant was inactive. On the other hand, the C4S version of MsrB1-Cys had activity similar to that of MsrB1-Cys in both DTT- and Trx-dependent reactions. Thus, Cys4 was required for the Trx-recycling process of the selenoprotein, but was not needed in the presence of DTT or for the Cys form of the enzyme.

We further tested the ability of the selenoprotein forms of MsrB2 and MsrB3 to catalyze Met-SO reduction in the presence of Trx. In contrast to the finding that these selenoproteins exhibited >100-fold increased activity in the presence of DTT compared to the natural Cys forms, these proteins were not active in the Trx-dependent assay (Table 3).

These data are consistent with the idea that a selenenic acid intermediate in natural or unnatural selenoprotein MsrBs could form a selenenylsulfide bond with the resolving Cys, which could then be reduced by Trx. However, in the absence of the resolving Cys, the selenenic acid intermediate could not be reduced by Trx, but was reducible by DTT. In fact, although the distance between Sec95 and Cys4 was calculated to be ∼10 Å in the MsrB1 structural model, the selenenylsulfide-bond formation should be possible because of flexibility of the N-terminal region of protein, which includes a hinge consisting of Gly8 and Gly9 (Figure S1B). In contrast, a sulfenic acid intermediate in Cys-containing MsrBs could either form a disulfide bond or be directly reduced by Trx or DTT.

**Figure 4:**
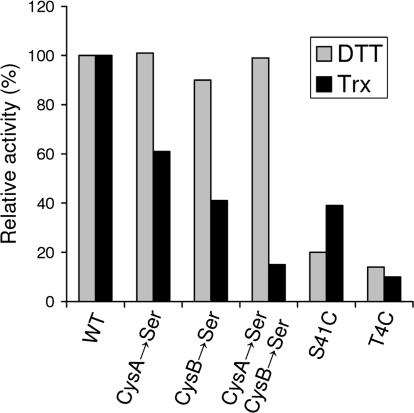
Relative Activity of Various Mutant Forms of MsrB3 Activities of the indicated mutants were normalized to those found in the wild-type form in both Trx-dependent (black bars) and DTT-dependent (gray bars) assays. Concentration of the substrate (dabsyl-Met-*R*-SO) was 200 μM in the DTT assay and 10 μM in the Trx assay. Proteins purified from *E. coli* were used. CysA and CysB correspond to positions 35 and 41, respectively, in the human MsrB3 sequence.

To test this possibility, two questions should be addressed. First, are there any resolving Cys residues in MsrB2 or MsrB3? Second, if no resolving Cys residues are present in these proteins, is it feasible to introduce an artificial resolving Cys, which could assist the selenoprotein forms of MsrB2 or MsrB3? To address the first question, we tested two Cys residues located in the N-terminal region (the only non-catalytic and non-zinc-binding Cys residues in MsrB3 sequences) for a possible role of a resolving Cys in MsrB3. These CysA and CysB residues correspond to positions 35 and 41, respectively, in the human MsrB3 sequence (highlighted in green in Figure 1C). These residues were mutated to Ser to generate single or double mutants. As shown in Figure 4, in the DTT assay, all single and double Cys→Ser mutants exhibited activities similar to that of the wild-type enzyme. In addition, all mutants were active in the Trx assay (even though the mutations decreased enzyme activities), suggesting that the resolving Cys was not required for MsrB3 function.

To answer the second question (concerning the introduction of an artificial resolving Cys in the selenoprotein forms of MsrB2 or MsrB3), we analyzed structural models and the multiple-sequence alignment (see Figure 1C) and selected two candidate sites for insertion of the candidate resolving Cys: (i) Ser41 in MsrB3 (Thr41 in MsrB2), which corresponds to the resolving Cys present in 60% of MsrBs; and (ii) Thr4 in MsrB3 and MsrB2, which corresponds to Cys4 in MsrB1. Separately, we mutated these residues to Cys in the seleno-MsrB3/C95U form and isolated proteins following large-scale transfections of NIH 3T3 cells. The activity of the S41C selenoprotein MsrB3 was slightly lower than that of the seleno-MsrB3/C95U in the presence of DTT (Table 3). However, this mutant had no activity in the Trx assay, as observed above for seleno-MsrB3/C95U. Although the T4C mutation decreased the MsrB activity 16-fold in the DTT-dependent reaction (compared to seleno-MsrB3/C95U), the T4C mutant form of this selenoprotein exhibited significant activity in the presence of Trx, which was as high as the activity of the natural Cys-containing form. Thus, the catalytic Sec/resolving Cys pair introduced into MsrB3 could fully replace the catalytic Cys in the Trx-dependent reaction. Since this effect was observed in the context of the active-site environment adapted for the Cys-containing enzyme, it is possible that this enzyme could be further improved by additional mutations in the active site (or by natural selection). These observations have direct implications for the possibility of improving catalytic properties of oxidoreductases containing catalytic Cys residues.

To test the effects of these mutations on the activity of the natural Cys-containing MsrB3, we generated S41C and T4C mutants of this protein in *E. coli* (see Figure 4). Both S41C and T4C mutations significantly decreased the enzyme activity. We also generated and assayed a T4C mutant of seleno-MsrB2/C95U (Table 3). The activity of this mutant in DTT assays was decreased 58-fold compared to that of seleno-MsrB2/C95U, and the mutant was not active in the presence of Trx.

**Figure 5:**
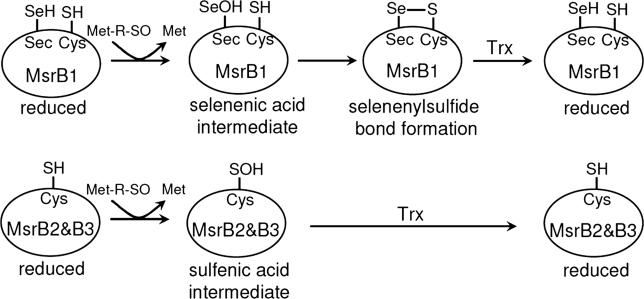
Models for Catalytic Mechanisms of Mammalian MsrB Enzymes In MsrB1 (upper part of the figure), the catalytic Sec directly attacks the Met-SO substrate. A resulting selenenic acid intermediate of selenoprotein MsrB1, which is not reducible by Trx, then forms a selenenylsulfide bond with resolving Cys4. Subsequently, the selenenylsulfide is reduced by Trx. In contrast, a sulfenic acid intermediate of MsrB2 and MsrB3 (lower part of the figure) can be directly reduced by Trx, making the resolving Cys dispensable.

Taken together, our data suggest that different catalytic mechanisms are employed by selenoprotein and non-selenoprotein forms of mammalian MsrBs, with the key difference being the reduction of proteins by Trx. As shown in Figure 5, a selenenic acid intermediate of selenoprotein MsrB1 could form a selenenylsulfide bond with the resolving Cys4. Subsequently, the selenenylsulfide is reduced by Trx. The selenenic acid intermediate itself, however, is not reducible by Trx. In contrast, the resolving Cys may be dispensable for Cys-containing MsrB2 and MsrB3, in which the sulfenic acid intermediate could be directly reduced by Trx. At least in MsrB3, introduction of the catalytic Sec/resolving Cys pair could change the reaction mechanism from that of Cys-containing mammalian MsrBs to that of MsrB1.

### Catalytic Advantages and Disadvantages of Sec-Containing Proteins Compared to Cys-Containing Counterparts

In a broader context, our data provide insights into catalytic advantages and disadvantages of Sec-containing proteins compared to their Cys-containing counterparts. It appears that Sec per se may often result in a higher catalytic activity, as illustrated in our study by a dramatic increase in the DTT-dependent reduction of Met-SO by Sec-containing MsrB2 and MsrB3 compared to their natural Cys-containing forms. It has previously been reported that the activity of a plant phospholipid hydroperoxide glutathione peroxidase is enhanced by simply replacing the active-site Cys with Sec [31]. However, the replacement of Cys with Sec may not only affect activity and substrate specificity, but may also completely change protein function. For example, a Sec-containing form of subtilisin became an efficient peroxidase rather than a protease [32]. The use of Sec in glutathione *S*-transferase also converted this protein into a peroxidase [33].

Selenoproteins typically are better catalysts than their Cys-containing orthologs. For example, the activity of Sec-containing formate dehydrogenases is much higher than that of the natural Cys-containing enzymes [34]. Thus, the enhanced activity of selenoproteins compared to their Cys-containing counterparts is an obvious advantage of Sec over Cys in certain types of protein function and is likely a reason why Sec evolves from Cys in proteins.

Does selenium play a role in increasing the catalytic activity of MsrBs only? We found that selenoprotein MsrB1 exhibited ∼2.5-fold lower activity than MsrB2 and MsrB3 in the presence of DTT (see Tables 1 and 2). However, in the Trx assay, the specific activity of selenoprotein MsrB1 was 4.5- and 2.0-fold higher than those of MsrB2 and MsrB3, respectively. Therefore, these data suggest that selenoprotein MsrB1 is a better substrate for Trx than Cys-containing MsrB2 and MsrB3.

Our data also identified catalytic disadvantages provided by Sec. Although the Met-SO reduction was increased more than 100-fold in the Sec-containing forms of MsrB2 and MsrB3, the regeneration of the active enzymes by the natural electron donor, Trx, was not possible. Some of our attempts to develop a highly active enzyme that could be reduced with Trx by introducing a resolving Cys did not succeed. However, the T4C form of Sec-containing MsrB3 was fully active (its activity was equal to that of the natural Cys-containing enzyme) in the Trx-dependent reaction. We propose that in MsrBs the use of Sec is a compromise between elevated rates of Met-SO reduction and the ability to regenerate the active enzyme form by reduction with the natural electron donor.

The regeneration of the Cys-containing proteins posed no problems as the sulfenic acid intermediate could be directly reduced by either DTT or Trx. It is possible that the reason why the insertion (at a place normally observed in Cys-containing MsrBs) of the resolving Cys in selenoprotein forms of MsrB2 and MsrB3 did not support regeneration of these proteins with Trx was a highly negative redox potential of the selenenylsulfide bond compared to the disulfide present in Cys-containing proteins. Selenenylsulfide is known to have a low redox potential [35]. Diselenide has an even lower redox potential, probably explaining why this group has never been observed in selenoenzymes.

### Evolutionary and Protein-Design Implications

As discussed above, it is highly likely that the selenoprotein MsrB1 evolved by the replacement of Cys with Sec. The conserved Thr4, which is identical in all non-selenoproteins, would have been concomitantly changed to Cys, which made the selenoprotein active in the Trx-dependent reaction. The conserved residues in the active site, such as His77, Val/Ile81, and Asn97 could then be replaced with Gly77, Glu81, and Phe97 to maximize enzyme activity.

The full advantages of Sec in proteins are probably not fully utilized because evolution of Sec is a difficult process. Not only must a Cys codon be changed to TGA (Sec codon), but the genes must evolve SECIS elements. At least in bacteria, where SECIS elements are located in coding regions, constraints imposed by protein sequence might not always be compatible with the evolution of a highly specific stem-loop structure of the SECIS element. In addition, Sec may evolve only in organisms which already have the Sec insertion system (approximately a quarter of all organisms) and only in environments where increased dependence on the trace element selenium may be satisfied. It is highly likely that the replacement of catalytic redox-active Cys with Sec in proteins may often enhance their activity, but its use can not be widespread.

Thus, evolution of Sec-containing proteins is a complex process, in which enhanced catalytic efficiency provided by Sec is in balance with limitations imposed by electron donors (or acceptors depending on the reaction), dependence on selenium, and the availability of the Sec insertion system.

It should be possible to apply these concepts to increase the catalytic efficiencies of various redox enzymes. Candidate strategies include direct replacement of catalytic Cys with Sec, as well as concomitant design of a resolving Cys and active-site environment. Enzymes containing catalytic redox-active Cys should be the best targets in order to design proteins with improved catalytic properties. For example, peroxiredoxins are viewed as important antioxidant proteins, but their Trx-dependent peroxidase activities are low compared to the activities of selenoprotein peroxidases, such as mammalian glutathione peroxidase. It is possible that selenoprotein peroxiredoxins, which are adapted for the use of catalytic Sec, will be superior catalysts. In fact, genes encoding bacterial Sec-containing peroxiredoxins have been detected.

In conclusion, this study shows the first evidence that the catalytic mechanism of selenoprotein MsrB differs from that of mammalian Cys-containing homologs with respect to the dependence on the unique resolving Cys and on the natural electron donor, and suggests that Sec- and Cys-containing proteins require a different set of active-site features that maximize their catalytic efficiencies. Based on these data, we propose that Sec per se may increase the catalytic efficiency of many oxidoreductases that utilize catalytic redox-active thiols, but the overall effect of having catalytic Sec should be viewed in the context of limitations that come with it.

## Materials and Methods

### Cloning, expression, and purification of wild-type and mutant forms of mouse MsrB1

An 810-bp cDNA for mouse MsrB1 including a 3′-untranslated region that contains the SECIS element [3] was cloned into *Xho*I/*Not*I sites of pCI-neo (Promega, Madison, Wisconsin, United States) to generate a construct that codes for the full-length Sec-containing MsrB1. In addition, we introduced five His residues at the C-terminus, which already had a C-terminal His. The resulting construct was named pCI-SelR-His. Through site-directed mutagenesis using QuickChange Site-Directed Mutagenesis Kit (Stratagene, La Jolla, California, United States), several constructs encoding various mutant MsrB1 forms with the C-terminal His tag were generated using pCI-SelR-His as template. All constructs were verified by DNA sequencing.

To express wild-type and mutant forms of MsrB1, the constructs were separately transfected into NIH 3T3 cells using Lipofectamine and Plus reagents (Invitrogen, Carlsbad, California, United States) on a large scale. The transfected cells were incubated in Dulbecco's modified Eagle medium containing 10% fetal calf serum, 0.6 μM sodium selenite, and antibiotics for 2 d at 37 °C under 5% CO_2_, collected by scrapping, washed twice with PBS buffer, and resuspended in PBS buffer containing 10 mM imidazole and 1 mM phenylmethylsulfonyl fluoride. Wild-type and mutant forms of MsrB1 were further purified using Talon metal-affinity resin according to the manufacturer's protocol (Clontech, Palo Alto, California, United States). The eluted proteins were concentrated and dialyzed against buffer A (50 mM sodium phosphate, pH 7.5, and 50 mM NaCl). Typical yield of the purified proteins was 2–7 μg from transfected cells grown in ten plates (diameter, 100 mm).

Various mutant forms of MsrB1-Cys containing an N-terminal His tag (MGSSHHHHHHSSGLVPRGSH), except for a C4S mutant, were generated through site-directed mutagenesis using the MsrB1-Cys construct [18] made on the basis of pET28a. To avoid possible interference resulting from the presence of the N-terminal tag close to the resolving Cys, we generated wild-type and C4S mutant proteins containing a C-terminal His-tag (LEHHHHHH) as follows. The cDNA of MsrB1-Cys was cloned into *Nde*I/*Xho*I sites of pET21b, resulting in plasmid pET21-MsrB1-Cys. Then, site-directed mutagenesis was carried out using the resulting plasmid as template. All constructs were verified by DNA sequencing. Wild-type or mutant forms of MsrB1-Cys were expressed in *E. coli* BL21(DE3) cells and purified using Talon metal-affinity resin (Clontech) as described previously [18]. The eluted proteins were dialyzed against buffer A, and analyzed for purity by SDS-PAGE. The enzyme activities of N-terminal and C-terminal tagged MsrB1-Cys were similar.

### Cloning, expression, and purification of wild-type and mutant forms of mouse MsrB2 and human MsrB3

Several constructs encoding mutant MsrB2 containing a C-terminal His tag (LEHHHHHH) were prepared through site-directed mutagenesis using a full-length mouse MsrB2 construct [18] as template. An MsrB3AΔ(1–31) construct [18] that lacked the endoplasmic reticulum–targeting sequence was used as template to generate various mutants of human MsrB3 containing a C-terminal His tag (LEHHHHHH). All constructs were verified by DNA sequencing. The constructs were expressed in *E. coli* BL21(DE3) cells, purified using Talon affinity column (Clontech), and dialyzed against buffer A. The purity of the purified proteins was analyzed by SDS-PAGE.

### Cloning, expression, and purification of selenoprotein forms of mouse MsrB2 and human MsrB3

A 457-bp cDNA fragment corresponding to the 3′-untranslated region of mouse MsrB1 and containing the MsrB1 SECIS element was cloned into *Sal*I/*Not*I sites of pCI-neo, resulting in plasmid pCI-SECIS. Mouse MsrB2(24–175) containing a C-terminal His tag (LEHHHHHH) and lacking the mitochondrial signal peptide was cloned into *EcoR*I/*Sal*I of pCI-SECIS to generate a wild-type construct, pCI-B2WT. A pCI-B2WT-Sec construct, which codes for a selenoprotein form of MsrB2, was generated by mutating the Cys162 codon (this position is based on the MsrB2 sequence) to the Sec codon, UGA. Other mutants of the selenoprotein MsrB2 form were made using pCI-B2WT-Sec as template by site-directed mutagenesis. Human MsrB3(32–192) containing a C-terminal His tag (LEHHHHHH) and lacking the endoplasmic reticulum signal was cloned into *EcoR*I/*Sal*I of pCI-SECIS to generate a wild-type construct, pCI-B3WT. To prepare a pCI-B3WT-Sec construct coding for a selenoprotein of MsrB3, a Cys158 codon (this position is based on the MsrB3 sequence) was replaced with a Sec codon, UGA. Other mutant forms of MsrB3 selenoprotein were made using pCI-B3WT-Sec as template by site-directed mutagenesis.

To express recombinant selenoprotein forms of MsrB2 and MsrB3, each of the constructs was separately transfected into NIH 3T3 cells on a large scale. For comparison, the constructs encoding Cys forms of proteins (pCI-B2WT or pCI-B3WT) were separately expressed in NIH 3T3 cells (see Table 3). Procedures for expression and purification of proteins were as described above for Sec-containing MsrB1.

### Determination of protein concentration

For all purified proteins expressed in *E. coli*, protein concentration was determined by the Bradford method using bovine serum albumin as a standard. Because of small amounts of the affinity-purified proteins expressed in NIH 3T3 cells, the protein concentration was determined by Western blotting with isozyme-specific antibodies using bacterially expressed recombinant MsrB1-Cys, full-length MsrB2, or MsrB3(32–192), as internal standards, followed by quantitation of the Western blot signals with a densitometer.

### Determination of MsrB activity and analysis of enzyme kinetics

MsrB activity was assayed in the presence of DTT or Trx. Dabsylated Met-*R*-SO was used as substrate. Catalytic activities of MsrBs with DTT were higher than with Trx. Different concentrations of the substrate in DTT- and Trx-dependent reactions were chosen to optimize the enzyme assays. In the DTT-dependent reaction, a typical reaction mixture (100 μl) for reduction of dabsyl-Met-*R*-SO to dabsyl-Met contained 50 mM sodium phosphate, pH 7.5, 50 mM NaCl, 20 mM DTT, 200 or 250 μM Met-*R*-SO, and purified proteins. In the Trx-dependent reaction, a typical reaction mixture (100 μl) contained 50 mM sodium phosphate, pH 7.5, 50 mM NaCl, 6.8 μM *E. coli* Trx (Sigma), 0.2 mM NADPH, 0.4 μM *E. coli* Trx reductase (Sigma), 10 μM substrate, and purified proteins. The reactions were carried out at 37 °C for 30–60 min and were then stopped by adding 200 μl of acetonitrile. The reaction product, dabsyl-Met, was analyzed by HPLC as described previously [26]. *K*_m_ and *k*_cat_ values were determined for the DTT-dependent reaction from Lineweaver-Burk plots.

It should be noted that the MsrB3 form expressed in *E. coli* exhibited an 11-fold higher activity than the proteins purified from NIH 3T3 cells. The reasons for this difference are not known. To avoid these variables, the proteins (including all controls) used in each experiment were prepared in the same expression system.

### Gel filtration

Apparent molecular masses of MsrB proteins were determined by gel filtration using a TSK-GEL G3000PWXL column (internal diameter 0.78 × 30 cm, Tosoh Bioscience, http://www.tosohbioscience.com). The recombinant proteins purified from *E. coli* were loaded onto the column and eluted with PBS buffer. The full-length MsrB1-Cys, full-length MsrB2, and MsrB3(32–192), were found to migrate as monomers.

### Molecular modeling

Structural models of mouse MsrB1 (residues 1 to 116), mouse MsrB2 (residues 36 to 175), and human MsrB3 (residues 42 to 184) were built using a crystal structure of the *N. gonorrhoeae* MsrB domain (PDB 1L1D) as template with 3D-JIGSAW (http://www.bmm.icnet.uk/servers/3djigsaw) [36] and SWISS-MODEL (http://swissmodel.expasy.org) [37], and the constructed models were evaluated with PROCHECK (http://www.biochem.ucl.ac.uk/bsm/biocomp) in CCP4 [38]. The modeled structures were visualized with PyMOL [39].

## Supporting Information

Figure S1 Structural Models of Mammalian MsrBs (A) Surface models of mouse MsrB2 and human MsrB3. Catalytic Cys95 residues are shown in red. Asn97 residues that sit at the bottom of the active-site pockets inMsrB2 and MsrB3 are shown in blue.
(B) Mouse MsrB1. In the structural model, the distance between Cys4 and Sec95 was ∼10 Å. A hinge consisting of Gly8 and Gly9 is shown in blue and indicated by arrows. Four Cys residues that coordinate Zn are shown in orange (the zinc atom is not shown).
(1.3 MB PDF).

### Accession Numbers

The GenBank (http://www.ncbi.nlm.nih.gov/Genbank) identification numbers for MsrB1, MsrB2, and MsrB3 sequences utilized in the study are 7305478, 27753987, and 72534836, respectively. The GenBank accession number for *D. melanogaster* Trx reductase is 10953878 and for *N. gonorrhoeae* PilB is 19526685. GenBank accession numbers for the amino acid sequences of MsrBs shown in Figure 1 (but not already listed above) are *Agrobacterium tumefaciens* (5888246), *Arabidopsis thaliana* (4115939), *D. melanogaster* (17944415), *E. coli* (15802192), *Helicobacter pylori* (3252888), *Hemophilus influenzae* (16273361), *Homo sapiens* MsrB1 (45439350), *H. sapiens* MsrB2 (20149599), *Methanothermobacter thermautotrophicus* (15678738), *Mus musculus* MsrB3 (15396336 and 31342784), *Mycobacterium tuberculosis* (15609811), *Saccharomyces cerevisiae* (6319816), *Salmonella enterica* (16760604), and *V. cholerae* (15642000).

## Abbreviations

Cys: cysteine
DTT: dithiothreitol
Met-SO: methionine sulfoxide
MsrA: methionine-*S*-sulfoxide reductase
MsrB: methionine-*R*-sulfoxide reductase
MsrB1-Cys: MsrB1 mutant in which Cys replaces Sec
Sec: selenocysteine
SECIS: selenocysteine insertion sequence
Ser: serine
Trx: thioredoxin

## References

[pbio-0030375-b01] Chambers I, Frampton J, Goldfarb P, Affara N, McBain W (1986). The structure of the mouse glutathione peroxidase gene: The selenocysteine in the active site is encoded by the “termination” codon, TGA. EMBO J.

[pbio-0030375-b02] Gladyshev VN, Jeang KT, Stadtman TC (1996). Selenocysteine, identified as the penultimate C-terminal residue in human T-cell thioredoxin reductase, corresponds to TGA in the human placental gene. Proc Natl Acad Sci U S A.

[pbio-0030375-b03] Kryukov GV, Kumar RA, Koc A, Sun Z, Gladyshev VN (2002). Selenoprotein R is a zinc-containing stereo-specific methionine sulfoxide reductase. Proc Natl Acad Sci U S A.

[pbio-0030375-b04] Jacob C, Giles GI, Giles NM, Sies H (2003). Sulfur and selenium: The role of oxidation state in protein structure and function. Angew Chem Int Ed.

[pbio-0030375-b05] Stadtman TC (1996). Selenocysteine. Annu Rev Biochem.

[pbio-0030375-b06] Hatfield DL, Gladyshev VN (2002). How selenium has altered our understanding of the genetic code. Mol Cell Biol.

[pbio-0030375-b07] Bock A, Forchhammer K, Heider J, Baron C (1991). Selenoprotein synthesis: An expansion of the genetic code. Trends Biochem Sci.

[pbio-0030375-b08] Gromer S, Johansson L, Bauer H, Arscott LD, Rauch S (2003). Active sites of thioredoxin reductases: Why selenoproteins?. Proc Natl Acad Sci U S A.

[pbio-0030375-b09] Weissbach H, Etienne F, Hoshi T, Heinemann SH, Lowther WT (2002). Peptide methionine sulfoxide reductase: Structure, mechanism of action, and biological function. Arch Biochem Biophys.

[pbio-0030375-b10] Weissbach H, Resnick L, Brot N (2005). Methionine sulfoxide reductases: History and cellular role in protecting against oxidative damage. Biochim Biophys Acta.

[pbio-0030375-b11] Petropoulos I, Friguet B (2005). Protein maintenance in aging and replicative senescence: A role for the peptide methionine sulfoxide reductases. Biochim Biophys Acta.

[pbio-0030375-b12] Stadtman ER, Van Remmen H, Richardson A, Wehr NB, Levine RL (2005). Methionine oxidation and aging. Biochim Biophys Acta.

[pbio-0030375-b13] Moskovitz J (2005). Methionine sulfoxide reductases: Ubiquitous enzymes involved in antioxidant defense, protein regulation, and prevention of aging-associated diseases. Biochim Biophys Acta.

[pbio-0030375-b14] Hansel A, Heinemann SH, Hoshi T (2005). Heterogeneity and function of mammalian MSRs: Enzymes for repair, protection and regulation. Biochim Biophys Acta.

[pbio-0030375-b15] Lescure A, Gautheret D, Carbon P, Krol A (1999). Novel selenoproteins identified in silico and in vivo by using a conserved RNA structural motif. J Biol Chem.

[pbio-0030375-b16] Huang W, Escribano J, Sarfarazi M, Coca-Prados M (1999). Identification, expression and chromosome localization of a human gene encoding a novel protein with similarity to the pilB family of transcriptional factors (pilin) and to bacterial peptide methionine sulfoxide reductases. Gene.

[pbio-0030375-b17] Jung S, Hansel A, Kasperczyk H, Hoshi T, Heinemann SH (2002). Activity, tissue distribution and site-directed mutagenesis of a human peptide methionine sulfoxide reductase of type B: hCBS1. FEBS Lett.

[pbio-0030375-b18] Kim HY, Gladyshev VN (2004). Methionine sulfoxide reduction in mammals: Characterization of methionine-*R*-sulfoxide reductases. Mol Biol Cell.

[pbio-0030375-b19] Kim HY, Gladyshev VN (2004). Characterization of mouse endoplasmic reticulum methionine-*R*-sulfoxide reductase. Biochem Biophys Res Commun.

[pbio-0030375-b20] Lowther WT, Weissbach H, Etienne F, Brot N, Matthews BW (2002). The mirrored methionine sulfoxide reductases of *Neisseria gonorrhoeae* pilB. Nat Struct Biol.

[pbio-0030375-b21] Lowther WT, Brot N, Weissbach H, Honek JF, Matthews BW (2000). Thiol-disulfide exchange is involved in the catalytic mechanism of peptide methionine sulfoxide reductase. Proc Natl Acad Sci U S A.

[pbio-0030375-b22] Lowther WT, Brot N, Weissbach H, Matthews BW (2000). Structure and mechanism of peptide methionine sulfoxide reductase, an “anti-oxidation” enzyme. Biochemistry.

[pbio-0030375-b23] Tete-Favier F, Cobessi D, Boschi-Muller S, Azza S, Branlant G (2000). Crystal structure of the *Escherichia coli* peptide methionine sulphoxide reductase at 1.9 Å resolution. Structure Fold Des.

[pbio-0030375-b24] Taylor AB, Benglis DM, Dhandayuthapani S, Hart PJ (2003). Structure of *Mycobacterium tuberculosis* methionine sulfoxide reductase A in complex with protein-bound methionine. J Bacteriol.

[pbio-0030375-b25] Antoine M, Boschi-Muller S, Branlant G (2003). Kinetic characterization of the chemical steps involved in the catalytic mechanism of methionine sulfoxide reductase A from *Neisseria meningitides*. J Biol Chem.

[pbio-0030375-b26] Kumar RA, Koc A, Cerny RL, Gladyshev VN (2002). Kumar RA, Koc A, Cerny RL, Gladyshev VN (2002) Reaction mechanism, evolutionary analysis, and role of zinc in Drosophila methionine-R-sulfoxide reductase. J Biol Chem.

[pbio-0030375-b27] Olry A, Boschi-Muller S, Marraud M, Sanglier-Cianferani S, Van Dorsselear A (2002). Characterization of the methionine sulfoxide reductase activities of PILB, a probable virulence factor from *Neisseria meningitidis*. J Biol Chem.

[pbio-0030375-b28] Olry A, Boschi-Muller S, Branlant G (2004). Kinetic characterization of the catalytic mechanism of methionine sulfoxide reductase B from *Neisseria meningitidis*. Biochemistry.

[pbio-0030375-b29] Kauffmann B, Aubry A, Favier F (2005). The three-dimensional structures of peptide methionine sulfoxide reductases: Current knowledge and open questions. Biochim Biophys Acta.

[pbio-0030375-b30] Neiers F, Kriznik A, Boschi-Muller S, Branlant G (2004). Evidence for a new sub-class of methionine sulfoxide reductases B with an alternative thioredoxin recognition signature. J Biol Chem.

[pbio-0030375-b31] Hazebrouck S, Camoin L, Faltin Z, Strosberg AD, Eshdat Y (2000). Substituting selenocysteine for catalytic cysteine 41 enhances enzymatic activity of plant phospholipid hydroperoxide glutathione peroxidase expressed in *Escherichia coli*. J Biol Chem.

[pbio-0030375-b32] Bell IM, Fisher ML, Wu ZP, Hilvert D (1993). Kinetic studies on the peroxidase activity of selenosubtilisin. Biochemistry.

[pbio-0030375-b33] Yu HJ, Liu JQ, Bock A, Li J, Luo GM (2005). Engineering glutathione transferase to a novel glutathione peroxidase mimic with high catalytic efficiency. Incorporation of selenocysteine into a glutathione-binding scaffold using an auxotrophic expression system. J Biol Chem.

[pbio-0030375-b34] Axley MJ, Bock A, Stadtman TC (1991). Catalytic properties of an *Escherichia coli* formate dehydrogenase mutant in which sulfur replaces selenium. Proc Natl Acad Sci U S A.

[pbio-0030375-b35] Besse D, Siedler F, Diercks T, Kessler H, Moroder L (1997). The redox potential of selenocystine in unconstrained cyclic peptides. Angew Chem Int Ed.

[pbio-0030375-b36] Bates PA, Kelley LA, MacCallum RM, Sternberg MJ (2001). Enhancement of protein modeling by human intervention in applying the automatic programs 3D-JIGSAW and 3D-PSSM. Proteins.

[pbio-0030375-b37] Schwede T, Kopp J, Guex N, Peitsch MC (2003). SWISS-MODEL: An automated protein homology-modeling server. Nucleic Acids Res.

[pbio-0030375-b38] Collaborative Computational Project (1994). The CCP4 Suite: Programs for protein crystallography. Acta Crystallogr D Biol Crystallogr.

[pbio-0030375-b39] DeLano WL (2002). The PyMOL Molecular Graphics System [computer program].

