# Supplementary material for: Different Catalytic Mechanisms in Mammalian Selenocysteine- and Cysteine-Containing Methionine-R-Sulfoxide Reductases
Source: PLoS Biol. 2005 Nov 8;3(12):e375. doi: 10.1371/journal.pbio.0030375 (PMC1278935; doi:10.1371/journal.pbio.0030375)

Figure S1

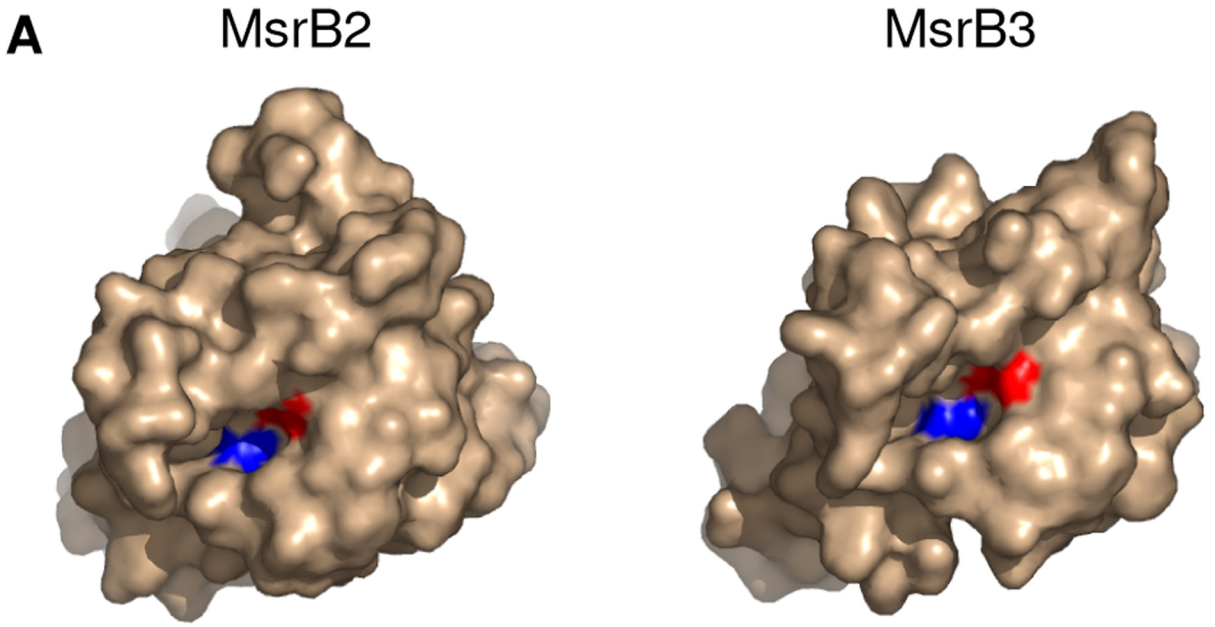

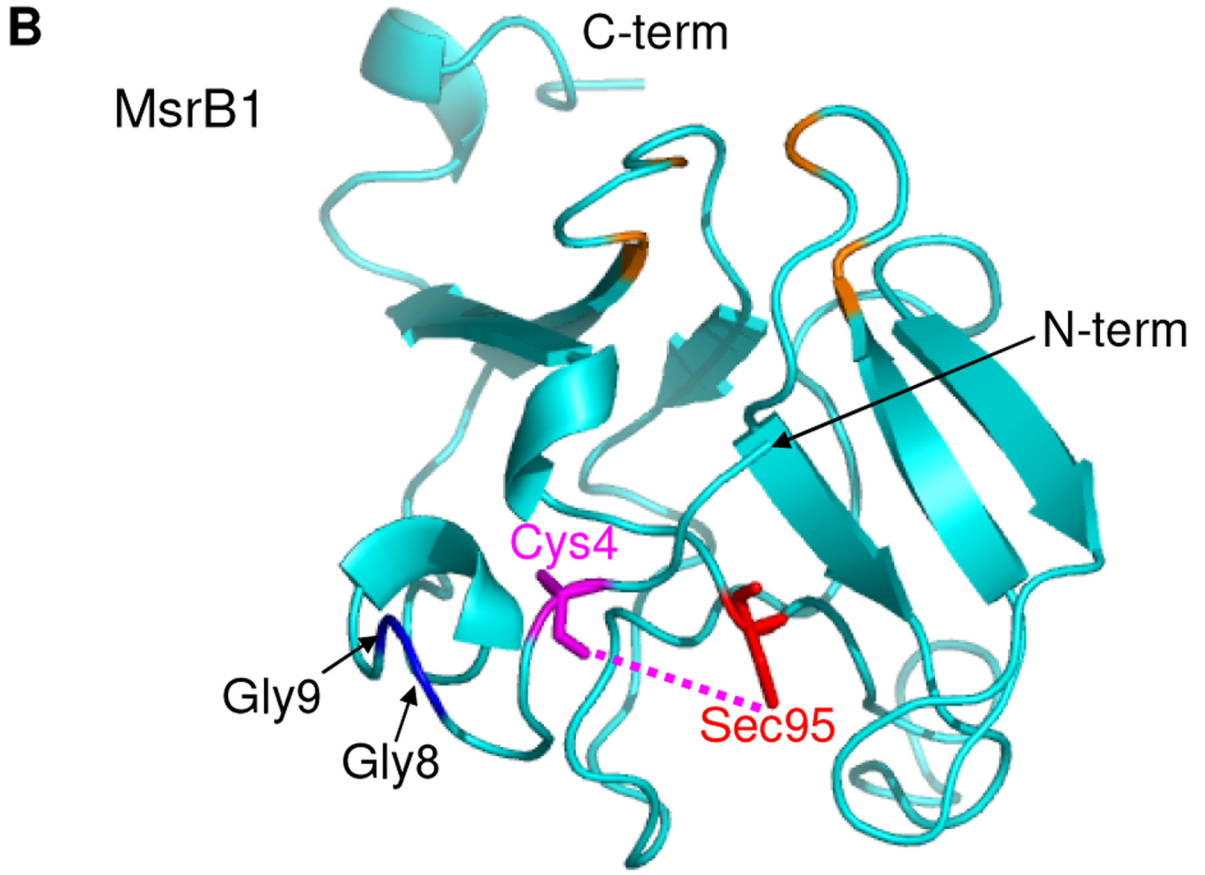

Supplement: Supplementary file 1 [file pbio.0030375.sg001.pdf]
